# Local Seal or Imported Meat? Sustainability Evaluation of Food Choices in Greenland, Based on Life Cycle Assessment

**DOI:** 10.3390/foods10061194

**Published:** 2021-05-25

**Authors:** Friederike Ziegler, Katarina Nilsson, Nette Levermann, Masaana Dorph, Bjarne Lyberth, Amalie A. Jessen, Geneviève Desportes

**Affiliations:** 1 RISE Research Institutes of Sweden, 40229 Gothenburg, Sweden; katarina.nilsson@ri.se; 2 Ministry of Fisheries, Hunting and Agriculture, 3900 Nuuk, Greenland; mail@nettelevermann.dk (N.L.); mado@nanoq.gl (M.D.); amalie@nanoq.gl (A.A.J.); 3 Association of Fishers and Hunters in Greenland (KNAPK), 3900 Nuuk, Greenland; bjarne@knapk.gl; 4 North Atlantic Marine Mammal Commission, 9091 Tromsø, Norway

**Keywords:** Greenland, greenhouse gas emissions, hunting, life cycle assessment, livestock, seal

## Abstract

Achieving a sustainable global food chain is becoming particularly acute as modern Western diets are adopted in a growing number of countries and cultures around the world. Understanding the consequences that this shift has on health and sustainability is important. This exploratory study is the first to apply the life cycle assessment (LCA) methodology to analyze the sustainability implication of ongoing dietary shifts in Greenland, where locally hunted seal meat is increasingly being replaced by imported livestock products, primarily pig and poultry produced in Denmark. This dietary shift, indirectly driven by international trade bans such as the EU seal product ban, has sustainability implications. To inform and support more comprehensive analyses and policy discussions, this paper explores the sustainability of these parallel Greenlandic food supply chains. A quantitative comparison of the greenhouse gas emissions of Greenlandic hunted seal and Danish pig and poultry is complemented by a qualitative discussion of nutrition, cultural food preferences, animal welfare, and the use of land, pesticides and antibiotics. Although the variability in the life cycle inventory data collected from Greenlandic hunters was considerable, greenhouse gas emissions of seal meat were consistently lower than those of imported livestock products. Emissions of the latter are dominated by biogenic emissions from feed production and manure management, while these are absent for seal meat, whose emissions instead are dominated by fossil fuel use. The implications of these results for sustainable national food policies in a modern global context as well as important areas for additional research are discussed.

## 1. Introduction

Food production is an important global contributor to resource use and environmental impacts, a role expected to become even more prominent in the future with a growing world population and changing dietary demands, as well as emission reductions in other sectors [1]. In recent years, the sustainability of food production, distribution and consumption has been scrutinized [2,3,4,5,6]. Various studies have begun to directly or indirectly build on and aggregate the growing body of food life cycle assessment (LCA) research, in which environmental impacts of food supply chains are quantified using a framework standardized by the International Organisation for Standardisation (ISO) [7,8]. Central environmental aspects in these LCA studies are greenhouse gas emissions, eutrophication and land use. Less frequently included but still critical aspects of the sustainability of food systems are toxic emissions (e.g., from the use of pesticides), animal welfare, and the entire social, cultural and economic dimensions of producing and consuming food. Recently published aggregations of food LCA research have shown that seafood, on average, leads to lower resource use and emissions than other animal source foods [2,4,5], but that seafood spans the entire range of other foods in terms of energy use [2,9]. When the nutritional content of seafoods is weighed in, it has been argued that seafood has clear benefits [10]. 

Seaweed and marine mammals are often left out of the definition of seafood [11], despite constituting critical dietary components and a significant resource potential in some parts of the world. Marine mammal consumption, for example, currently provides economic benefits in 54 out of 195 countries [12]. In terms of nutrition, these two resources have very special—and opposite—characteristics. While seaweeds contain many essential micro-nutrients, there are questions about their bioavailability [13]. The meat of marine mammals, on the other hand, is rich in many of the micro-nutrients typical for seafood (e.g., omega-3 fatty acids, vitamins B12, D, niacin, selenium, iodine and iron) in highly bioavailable forms [14,15]. However, some marine mammals are found at the top of marine food webs and bioaccumulate environmental pollutants such as heavy metals and organic compounds (released from industrial processes through air and water) at potentially hazardous concentrations, even very far away from the emission source [16]. However, dietary advice in countries where marine mammals are regularly consumed, e.g., Greenland, still often recommends following a traditional diet because the benefits are considered larger than the risks [17,18,19].

In Greenland, Inuit culture has traditionally been, and in some places still is, more based on subsistence hunting than on fishing, which has been more of a complementary activity. Marine mammals have been more accessible with indigenous hunting methods than the most important fish resources available (Greenland halibut and shrimp), which are found in deeper waters [20] and could not be exploited until industrial fishing methods were introduced from Europe. With extremely limited access to agricultural land, permafrost and a harsh arctic climate, the diet of Greenland Inuit has historically been based on protein and fat, mostly from the sea. In fact, the Inuit have genetically adapted to this diet and their environment to utilize protein and fat more efficiently [21]. In the present day, there is still a widespread dietary preference for traditional Greenlandic food, Kalaalimerngit, over other types of food, which are almost exclusively imported.

From a population of 56,000, around 7000 Greenlanders are hunters, either professional (around 2000) or leisure hunters, hunting game, whales, walruses and seals for food and clothing. The four hunted seal species are harp seal (*Pagophilus groenlandicus*), ringed seal (*Pusa hispida*), hooded seals (*Cystophora cristata*) and bearded seal (*Erignathus barbatus*), while grey seal (*Halichoerus grypus*) and harbor seal (*Phoca vitulina*) are protected. Seal hunting in Greenland is practiced using two main methods, depending on species, region and season [22,23,24]: shooting seals with a rifle or catching them under the ice using a net (predominantly in the north and east during winter months). Shooting can be further divided into two main strategies, defined by how the hunter moves: from a dinghy (small boat) or on the ice by foot, dogsled or snowmobile. 

**Figure 1:**
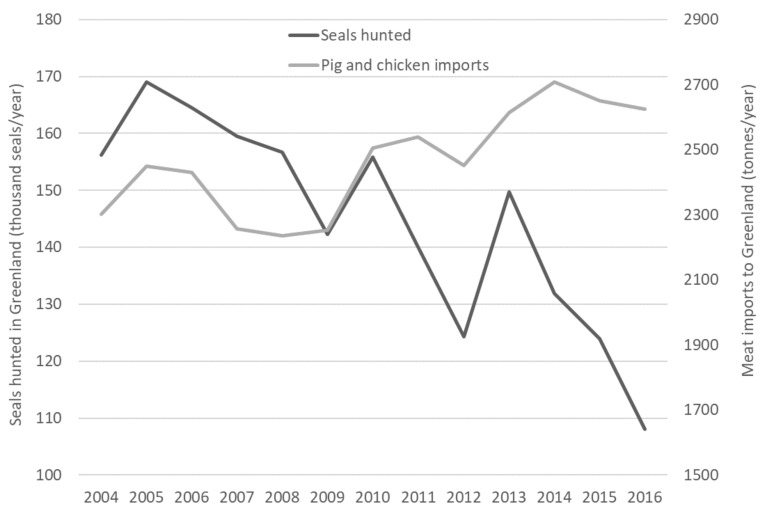
Seal hunting and imports of livestock products to Greenland between 2004 and 2016. Data from *Piniarneq* hunting database at the Ministry of Fisheries, Hunting and Agriculture and Statistics Greenland (www.stat.gl, accessed on 26 March 2021).

Recently, sealing in Greenland has decreased significantly (Figure 1, data from *Piniarneq* hunting database). This can be seen as an indirect consequence of the EU seal regime policy to ban the trading of commercial seal products, including skins [25]. Although there are exceptions for products from Inuit communities to the ban, the public perception of seal products has changed dramatically since the campaigns of the 1970s and the ban has reduced the European market and demand for seal products significantly. 

Seal and game meat are still consumed by hunters in Greenland and sold in local open markets, Kalaaliaraaq. It is, however, the seal skins that are the commercially valuable by-product that compensate for the cost of hunting activities (e.g., for the use of fuel and ammunition). The recent decrease or total lack of income from seal skins renders seal hunting an economically unviable activity for many Greenlanders. Diets have therefore shifted towards imported livestock products, such as chicken and pig meat (Figure 1). An unintended side effect of the policy to ban commercial seal products in Europe has therefore been a shift away from the consumption of locally hunted seal meat in Greenland and towards imported products. 

Here, we evaluate the environmental implications of this shift in Greenlandic animal-source food consumption and perform a first exploratory analysis of the sustainability of locally hunted seal meat vs. imported livestock products. We do this by using the life cycle assessment (LCA) method to quantitatively compare the greenhouse gas emissions of the alternative supply chains and qualitatively discuss a number of additional aspects relevant for supply chain sustainability assessment. The results of this work point to the role food policies play in influencing dietary shifts and the sustainability of the system. While this research arguably only represents an exploratory step in the comprehensive analytical work required to understand the sustainability of global food chains, the intention is to demonstrate the importance of comparing alternative food sources in shifting diets and to point to future research needs.

## 2. Materials and Methods

In this research, we applied the life cycle assessment (LCA) methodology to the case of Greenlandic seal hunting, comparing it to the most common alternative livestock products consumed in Greenland, pig and poultry meat imported from Denmark. Below, we present the specific methodological choices made in this study; for a more general introduction to LCA, see [26,27]. The use of LCA has become a regular practice to support the implementation of environmentally oriented decisions at a cooperative and/or political level in the US and in Europe [28].

### 2.1. Goal and Scope 

In this study, our unit of comparison is a kilo of meat in a Greenlandic household.

**Figure 2:**
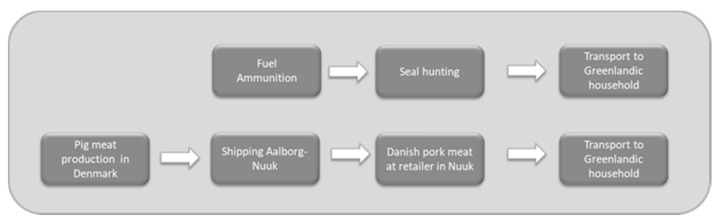
The two main supply chains for consumption of animal source foods in Greenland. The transport to the household was not included in the study but was modelled in the sensitivity analysis.

For seal meat, the system investigated entails the production and use of supply materials used during hunting (gasoline and ammunition) (Figure 2). The production of infrastructure with a lifetime longer than one year (such as rifle, boat, dock, etc.) was excluded. We obtained data on resource use for seal hunting through the Association of Fishers and Hunters in Greenland, KNAPK, who distributed a survey among their members. The survey contained questions about how the hunting is performed, transport to and from the hunt, the annual number of seals shot, amount of ammunition and fuel used, etc. From this survey, we obtained first-hand data from eleven professional hunters, who were mainly hunting ringed and harp seals and primarily using rifles, but also with nets, on occasion. It should be noted that bearded and hooded seals, walruses, minke and humpback whales were also sometimes hunted on the same occasions. We also obtained data from two of the major suppliers of ammunition, fuel and nets, who provided specific details regarding the ammunition (materials, import routes) and type of fuel used. It was decided to model the livestock import supply chain only to the port of Nuuk, meaning that the last transport to the household (Figure 2) is not included. The decision was based on difficulties to define an average or typical scenario for this transport, which could be long- or short-distance, take place by sea or air. No domestic transportation was included for the seal meat either and the importance of this omission on the results and conclusions is tested in the sensitivity analysis. 

The alternative food sources were determined studying import statistics from Statistics Greenland and consulting Greenlandic retailers, both indicating that pig and poultry are the dominating types of meat and that they are primarily sourced from Denmark. We investigated transportation routes and modes for food imports to Nuuk by talking to our contacts in Greenland, four Greenlandic food retailers as well as food exporters in the Nordic countries and added this transport route (shipping on a refrigerated containerized freight ship, 4931 km from Aalborg, Denmark, to Nuuk) to the farmgate data for Danish pig and poultry production, as presented in [29,30]. Leip et al.’s model of European livestock production through a cradle-to-gate LCA using the Common Agriculture Policy Regionalized Impact (CAPRI) model to estimate/calculate emissions. We included direct emissions due to land use, but excluded those resulting from indirect land use change, as modelled by Leip et al. [29,30] due to the large uncertainties in the underlying method to estimate these emissions and in order to not overestimate livestock emissions. However, as a result, livestock emissions are arguably underestimated.

### 2.2. Impact Assessment and Allocation

The environmental impact category studied in this exploratory study is greenhouse gas emissions (GHGs). Aggregate GHGs are also referred to as the global warming potential, climate change, climate impact or carbon footprint. The sustainability of marine resource use, land use, toxicity, welfare, nutritional content and food preferences are discussed qualitatively.

**Figure 3:**
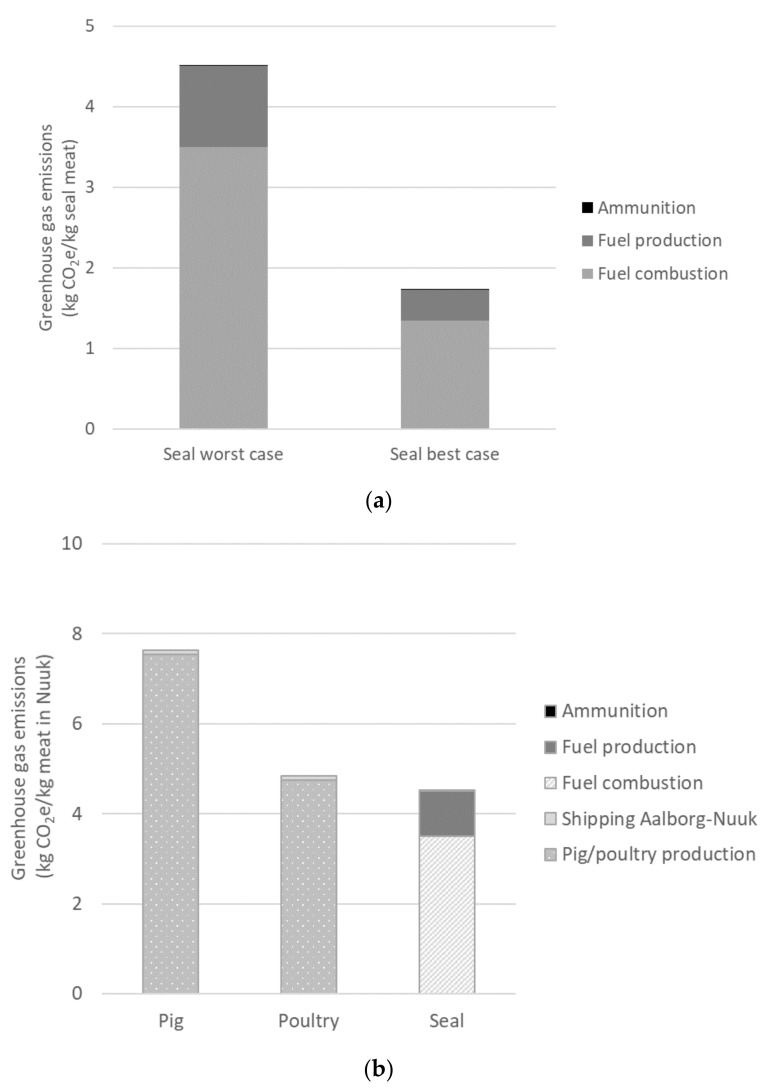
Greenhouse gas emissions of (**a**) hunted seal meat with fuel use modelled either based on effort, i.e., boat engine hours (worst case) or on reported annual fuel use of hunters (best case) and (**b**) imported pig and poultry (supply chains starting with feed production, pig/poultry production and shipping to Nuuk, Greenland) and locally hunted seal meat (worst case). Data for pig and poultry from [29,30].

When more than one product arises from a process (e.g., at slaughter and subsequent processing of seal), no use of by-products was assumed and all impacts of hunting were attributed to the edible food product. We think that the goal of the study, which is to compare the two production systems and supply chains from a food production perspective, justifies this choice. If one of the products renders much less food per biomass produced, allocating impacts to the non-edible by-products would be beneficial for this product, which would be inappropriate for the comparison. During the hunt, resource use has been allocated to all animals hunted on one occasion and this contributes to variability between hunters and hunting occasions. For the livestock products, however, the burden was split between products and by-products based on their relative economic value in the study utilized, which could not be changed. This is another method difference favoring livestock, as a part of the impact of agriculture is attributed to the by-products in the case of pig, but not in the case of seal meat. The two supply chains are shown in Figure 3.

Data was modelled in LCA software SimaPro MultiUser version 8.5.2. Background data for the production of materials and energy and for transport was taken from the life cycle database ecoinvent 3.4. Characterization factors for greenhouse gas emissions were based on [31].

## 3. Results

### 3.1. LCA Inventory and Impact Assessment Results

Fuel use was reported in two different ways by the hunters. The first strategy was to ask for the annual fuel use and what the hunting outcome was from using that amount of fuel. The second strategy was to ask how much fuel the engine uses per hour, how many hours per day and days per year the boat operates and what animals are hunted during the same period. For some, the former was easier to answer, whilst for others, the latter. The latter, “effort-based” approach led to a considerably larger fuel use estimate (1.54 ± 1.80 L/kg seal meat) than the one based on annual fuel use (0.60 ± 0.24 L/kg seal meat) resulting in greenhouse gas emissions of hunted seal meat that were more than double (Figure 3a). It is difficult to say which approach to calculating fuel use is more accurate. Ideally, they would show the same result and serve as a validation of each other [32]. The large variability arguably originates from the not clearly defined nature of the activity. For example, hunting trips are often combined with other activities and are not undertaken in a standardized way. In addition, the size and age of boats and engines varies, as do distances travelled to the hunt. Therefore, to be able to contribute to the study, hunters had to make estimations, with various sources of error contributing to the large spread. Hunters reported using 1 to 10 bullets per seal hunted, resulting in the average use of around 4 g of ammunition per kg of seal meat. Data on edible yield for the various marine mammals hunted was available from the Government of Greenland [33]. For ringed and harp seals, the average edible yields were 20 to 60 and 75 to 100 kg of meat per seal, respectively. Seal by-products are often used as dog food, but this was, as mentioned, not accounted for. The worst-case scenario (the effort-based approach to fuel use, Figure 3a) is the one further used in the comparison with imported livestock.

Following the analysis, the emissions for the worst-case scenario seal meat (4.5 kg CO_2_e/kg) are lower than those for pig meat (7.6 kg CO_2_e/kg) and marginally lower than that of poultry (4.7 kg CO_2_e/kg). Emissions of livestock products were revealed as mainly generated in the production phase, not due to the shipping to Greenland (Figure 3b). In the production phase, emissions consist more of biogenic emissions (methane and dinitrogen oxide) from manure management and soil management than from fossil energy use. Note that fuel production and combustion (the categories shown for sealing) are included in several sub-processes of livestock production, whereas in sealing these are the two dominant activities. The emissions of Danish pig production (the fifth largest producer of pig meat in the EU) are close to the average of EU-27 member states [29,30] and data provided by Leip.

### 3.2. Qualitative Results for Other Categories

In addition to greenhouse gas emissions, a number of additional aspects are relevant for considering the sustainability of the two animal food source production systems (ranked in Table 1). While these aspects cannot be addressed in detail here, references are provided for where more detailed information is available and it is important for this exploratory study to highlight and outline these as additional relevant factors to account for in more comprehensive assessments.

**Land use**: Danish pig and poultry production requires a land area of 0.67 ha/kg of meat, mainly for feed production (data extracted from the database behind Leip et al. [29,30], while seals live in the wild and do not need any input of manufactured feed or a designated area for their production. This makes the seal production system significantly less likely to contribute to destructive land use, which is a major environmental impact of agricultural systems [34]. 

**Animal welfare**: from an animal welfare perspective, there is a heated debate as to whether seal hunting can be practiced in a humane way [35,36,37,38,39]. Despite the European ban on commercial seal products, the European Food Safety Authority (EFSA) has in fact concluded that seals can be killed rapidly and effectively without causing avoidable pain, distress, fear and other forms of suffering, both using clubbing and shooting [40]. However, netting is not considered a humane killing method, as death by suffocation and drowning is protracted. Therefore, we chose to differentiate the two killing methods in our ranking of these additional factors below (Table 1). Animal welfare challenges in modern industrial pig and poultry production are well-known and documented [41,42,43,44], including skeletal problems and feather pecking among chicken and piglet mortality, tail biting, and aggression in group-housed sows. The welfare challenges of pig and poultry production occur throughout the entire lifetime of the animal and are therefore very different from those of seal hunting, in which questions of welfare arise only at the moment of killing. 

**Marine resource utilization**: in terms of the sustainable use of marine resources, the stock status of harp and hooded seal stocks are regularly assessed by the ICES/NAFO/NAMMCO Working Group on Harp and Hooded Seals [45]. Both harp seals and ringed seals, the two main seal species hunted in Greenland (around 57,000 and 48,000 seals hunted in Greenland in 2016) are listed as being of ‘least concern’ both on the recent global [46] and the Greenlandic Red List [47], the former species showing increasing population trends. This means that there are currently no concerns regarding the abundance of these seals [22,23]. The hooded seal is ranked as ‘vulnerable’ on both lists (around 1500 seals hunted in Greenland in 2016). The bearded seal is ranked ‘least concern’ on the global Red List and ‘data deficient’ on the Greenlandic Red List (around 1300 seals hunted in 2016). Walrus, which was also caught by the hunters, is ranked ‘vulnerable, but is under a quota regime informed by regular surveys and assessments (last in 2018) and following advice from [48]. Marine feed inputs used in piglet and poultry feeds only partly originate in sustainable fisheries.

**Nutrition**: the nutritional content of seal meat is, as already mentioned, high, and nutrients are found in a highly bioavailable form. While being a good source of protein, pig meat contains fewer desirable nutrients (omega-3 fatty acids, minerals and vitamins) and more undesirable nutrients (such as saturated fats). Poultry has a lower content of both desirable and undesirable nutrients [10]. 

**Contaminants**: conventional pig feeds are produced using various types of pesticides, which are then spread to environmental systems and organisms through the air and water. Among the food products studied by Nordborg et al. [49], pig and poultry caused the highest freshwater toxic emissions, mainly due to the extensive use of pesticides in Brazilian soy farming, acommon component of feed). Toxic emissions related to seal hunting mainly originate from the production of metal for the ammunition, from the production and combustion of fuel, and potentially from the use of anti-fouling paints on dinghies. While the risk of contamination with environmental pollutants is higher for marine mammal meat than pig meat [14,50], particularly for certain species, including seals, and certain organs (kidney, liver and fat), antibiotics used in the production of pigs and traces of veterinary medicines cannot be found in marine mammal meat.

**Food preferences**: the preference of Greenlanders for traditional over Western diets is well-documented, e.g., by Hansen et al. [14], and is a relevant aspect to consider if the social dimension of sustainability is to be taken into account. It is linked to maintaining traditional ways of life and livelihoods, which has both welfare and identity dimensions, but also an economic one in places where there are few alternatives to hunting in terms of making a living.

## 4. Discussion

Based on the data available, for Greenlandic people, seal meat from hunting in Greenland appears to be a more sustainable and environmentally preferable animal food option than imported livestock products, from a climate perspective, and in terms of a number of qualitatively assessed aspects. This result was despite making several assumptions and methodological choices favoring livestock in the LCA. However, the results do rest on limited data and further research on this topic is needed to deliver a more comprehensive analysis. 

Few types of food are as stigmatized within environmental circles as marine mammals, (including seals) and as ignored as a potential food resource in the general discussion of food security [11,51,52]. Current EU policy bans on trading sustainably hunted seal products based on the undefined and vague term “moral concern” seem unjustified when considered in light of EFSA’s determination regarding humane killing, the moral concerns linked to industrial animal agriculture and the lack of bans limiting the trade of seafood originating from overexploited stocks [53]. Recognizing the differences in the environmental consequences of different food sourcing systems in different locations is essential for creating sustainable global food chains. For people living in Greenland, this first exploratory study indicates that under the prevailing assumptions and policies, it is a more sustainable food choice to eat locally hunted seal meat than imported pig or poultry products. If this is indeed the case, it would also be best to maximize the utilization of the resource by making use of and trading the by-products, such as the skins. 

In livestock production, a large part of the emissions is biogenic and related to agricultural practices, including both feed production and manure management on the farm. There are ways to reduce these emissions [29], which could shift the outcome of the comparison. However, it is also important to note that we made several methodological choices in our LCA analysis that favored livestock production and still it did not perform better. The most important choice that we made in this regard was to exclude indirect land use change impacts altogether. The emissions from these impacts represent around 2.5 kg CO_2_e per kg of the average EU poultry and pig produced, representing 35 and 25% of total emissions, respectively [29]. The decision to exclude this part was based on the considerable uncertainty reflected by the range of values provided in the study. However, it is clear that livestock emissions would be considerably higher if these emissions had been included. Another aspect favoring livestock compared to seal meat was the fact that for seal, we decided to place all emissions on the meat, whereas economic allocation is carried out when converting carcass weight to edible meat, which means that pig by-products carry a part of the environmental burden of the production system, although a small part. Additionally, we did not model the domestic transport of pig meat to Greenlandic consumers outside Nuuk. This decision was based on a sensitivity analysis that suggested that it would only increase emissions slightly, since, according to our sources for import logistics, an overwhelming part of these domestic transports (97%) are made by ship, and only around 3% by air. 

What lessons have we learned from this exploratory assessment of impacts of everyday food choices in Greenland? Below, we discuss some improvement options that can be identified based on the one parameter that was analyzed quantitatively, greenhouse gas emissions. The use of fuel clearly dominates the greenhouse gases in seal hunting; hence, the main improvement potential lies in reducing this factor, which also lies in the direct interest of the hunters since fuel represents an important cost. Fewer, more dedicated hunting trips might be something to strive for, in cases when seals are found to be more aggregated on the sea ice. Less use of boats, possibly also other types of fuel or even electric outboard engines would reduce emissions, but the process of climate change itself, causing reduced sea ice cover, likely contributes to shifting Greenlandic seal hunting from ice to water. To be able to optimize production and minimize negative impacts, improved knowledge on the variability between hunting methods, seasons, species and particular practices (hunting on ice or from water) is important, as this knowledge could help inform seal management in Greenland. Seals are part of the marine ecosystem and it is therefore important to consider the impact on the ecosystem as a whole in the management of seals, and, e.g., take into account interactions with commercially fished species from changed seal hunting patterns [54,55].

Given that this exploratory study only compared two different sources of animal protein, a justified question could be: could Greenlanders reduce their greenhouse gas emissions by eating a vegetarian diet? It should be noted that this source of protein would also need to be imported, as the agricultural areas of Greenland are small, and the growth season is short, this would therefore not contribute to either food sovereignty or economic sustainability for Greenlanders. For a population used to, and even genetically adapted to, consuming mostly marine and terrestrial mammal meat [21] with a high nutritional value [14,15], it would also be a large step to switch to entirely vegetarian food. In addition to challenges of consumer acceptance and the specific nutritional needs of the Greenlandic population, the bioavailability of critical nutrients may also be higher in animal-sourced foods [14,15]. In addition, many settlements along the coast are only visited by ships once or twice per year, and people would therefore be limited to canned products, which, due to the preservation and packaging method, are known to have a high climate footprint, even when imported by sea. The long coastline of Greenland invites thinking about coastal seafood, including mussels and algae, potentially playing a larger role in the future diet. Indeed, more comprehensive analyses of different food options and choices for isolated communities such as those in Greenland would be informative for understanding how to achieve sustainable food chains. 

The results of this study can also be used to propose some improvement options for some of the parameters that were analyzed qualitatively, such as animal welfare. There are certainly animal welfare challenges in seal hunting, as in any hunt of wild game. This includes reducing the ratio of ‘struck and lost’ during the hunt, i.e., animals that are injured but not caught. This is not only a welfare problem but also contributes to increased greenhouse gas emissions, as it can be considered a loss of the hunt, similar to how emissions increase when animals are lost, e.g., due to disease in other production systems. There might be ways to minimize the struck-and-lost ratio, e.g., through a closure of the hunt when the water salinity is lowest due to melting snow and ice (May to June) and the animals do not have a thick layer of fat that makes them more buoyant—two factors contributing to a higher struck-and-lost rate during that period [22,23], but such measures would limit the food supply in some areas, which would have to be taken into account. Another way to reduce the struck-and-lost ratio (while improving hunter safety) would be to always have two hunters hunting together, where one is ready to shoot if the other one misses and to retrieve the sinking seal. Netting of seals is a hunting method that makes use of the development of gillnets in fisheries which started being used in the 1950s. It is only permitted in certain areas where it has made it possible to hunt also during the dark winter months. Although netted seals are rarely lost and have a minimal struck-and-lost rate, netting of marine mammals is potentially a more severe animal welfare issue than shooting [40]. Hunting methods should always be optimized as much as possible regarding animal welfare and hunters’ safety [56,57] and it is the practice of the hunt, i.e., which animal is hunted in which area by whom, that defines a traditional activity, not the tool used for hunting. 

Seal hunting with rifles, if performed following guidelines and best practice, is not different from game hunting, which is widely accepted. The killing of mammals is also widely accepted in livestock agriculture, and the animal welfare challenges in pig and poultry production, as mentioned earlier, are numerous, well-documented and span the entire lifespan of the animals [41,42,43,44]. Based on this knowledge and EFSAs conclusion that seals can be killed effectively [40], the total banning of seal hunting does not appear to be grounded in consistent consideration of animal welfare. 

The ban on sales of seal products in the European Union in 2009 [25], which has an exemption for Inuit-hunted seals, was not based on clearly documented conservation or animal welfare concerns, but rather on a “moral concern”. This can be interpreted as giving marine mammals a special status and that they should not be killed for any reason, irrespective of the method. This policy followed decades of campaigns of NGOs in Europe and North America (e.g., Greenpeace and IFAW) against sealing, exemplified by pictures of seal pups who were clubbed for their fur. Neither clubbing of seals nor hunting of seals only for the fur is legal or practiced in Greenland. Still, the ban led to an implosion of the market for seal products and many hunters had to stop hunting seals when there was no income from the skin and shifted to fisheries where there were better economic prospects. In this way, the seal product ban has had severe negative consequences for Arctic Inuit communities. Greenpeace have expressed their regrets and even apologized for their contribution to this development [23], but today, several other groups/NGOs are still running anti-sealing campaigns. The WWF has suggested that the EU should take an initiative to certify Inuit seal products in order to repair some of the damage it has caused to Greenlandic Inuit communities [23] and fair information campaigns are also advocated by Inuit Sila, Government of Greenland [58] and Rasmussen [59]. 

In an effort to compensate for the loss of income and support work opportunities in small hunting communities, since 2008, the Greenlandic government has subsidized the trade in sealskin by reimbursing Great Greenland A/S (the Greenlandic tanning company) the value of the skin. However, many of the skins are not exported, but now kept for the national market because the global demand for fur constantly changes. Additionally, the subsidized price paid for the skins has become so low that it does not pay the expenses of hunting and the labor of preparing them, so the quantity of skins sold to Great Greenland A/S is decreasing to a level where the tannery is no longer able to reach the target of the contract with the government. It is therefore noteworthy that while seals are still killed and consumed by humans and by sled dogs, the seal ban induced the waste of a by-product, the skins. 

Marine resources are the basis of the blue bioeconomy, which is strongly promoted on all levels, from local/regional to national/international, e.g., on the European level [60]. Large-scale future potential for a sustainable blue bioeconomy is identified in smarter utilization to maximize the food and economic value generated from sustainable use of marine living resources. The strategies, when speaking of marine living resources, are, however, typically limited to fisheries and aquaculture and by-products from these streams, and do not mention the utilization of marine mammal resources as a future potential [60]. Our exploratory study suggests that the reasons for this require further elaboration and the potential sustainability of marine mammal use further analysis and consideration. 

The only previous estimate of greenhouse gas emissions of marine mammal meat we are aware of is one calculated on the basis of eight Norwegian commercial whaling vessels in 2008 [61]. The average emissions were 1.9 kg CO_2_e/kg of minke whale meat (min 0.7 and max 2.7). The method used differs slightly from the method used in our study and if we apply the same method, emissions are slightly higher, 2.1 kg CO_2_e/kg of whale meat. These values are lower than the worst-case values found for Greenlandic sealing, but close to the best-case values we found and suggest that the hunting of marine mammals, whether for subsistence or commercial purposes, does not give rise to higher emissions than many other animal source foods. Studies of land-based hunting have similarly found that meat from wild boar can have lower emissions than most other animal source foods [62]. Ruminants such as deer, however, give rise to higher emissions [62], due to low productivity in combination with methane emissions from ruminant animals. Saxe [63] came to opposite conclusions about wild boar vs. pig and deer vs. beef, but had excluded feed digestion and manure management, which often constitute important parts of the footprint of animal source foods; the study, therefore, cannot be said to give a full picture of emissions.

Although the main policy considered in this work has been the EU ban on seal products, other policy areas may also learn from the results. Although representing a case study from Greenland, the work has potential implications for all Arctic nations and populations in which the utilization of marine mammals and other local food sources is part of the culture and economy. Potential policy improvements suggested from this work include:

1. A clear articulation of the goals of the EU seal product ban, as well as an evaluation of its underlying motives, direct and indirect consequences.
2. Greenlandic seal hunting regulations should further strive to minimize suffering of animals, e.g., by evaluating, and when needed improving or phasing out methods, potentially not living up to this standard as well as introducing measures that would further minimize the number of seals that are struck and lost. It should be taken into account that such regulations potentially affect the food supply of remote communities.
3. An evaluation of whether changes should be made in Greenlandic food policy, such as dietary advice or other tools stimulating and promoting a healthy and sustainable diet for Greenlanders.

To create sustainable global food chains, it is essential that consumer choices, and the food policies that help shape the options available, are well-informed by knowledge about the relative environmental and societal costs of different alternatives. It is therefore crucial that exploratory studies such as this one continue to be conducted in different locations around the world and that these are extended into ever-more comprehensive analyses as knowledge continues to be gained.

## Figures and Tables

**Table 1:** Qualitative summary of findings from analysis and literature on relevant aspects of the supply chains studied; green = advantage, yellow = small risk or lack of knowledge, red = disadvantage.

Category	Danish Poultry	Danish Pig	Greenlandic Seal, Rifle	Greenlandic Seal, Net
Greenhouse gas emissions ^1^				
Land use				
Animal welfare				
Marine resource use sustainability ^2^				
Nutrition				
Pesticide use				
Contaminant risk ^3^				
Antimicrobial use				
Food preference				

^1^ Excluding emissions from land use change which would affect pig and poultry, but not seal. ^2^ Greenlandic seal populations are sustainably harvested, fish used for fish meal for poultry/piglet feeds only partly. ^3^ The Greenland Board of Nutrition recommends continuing to eat traditional food but advises children, young and pregnant and nursing women against the consumption of older seals, toothed whales, sea birds and polar bears, due to the high level of contaminants [17].

## Data Availability

Life cycle inventory and impact assessment data is available upon request from corresponding author.

## References

[1] Searchinger T., Waite R., Hanson C., Ranganathan J., Dumas P., Matthews E. (2018). Creating a Sustainable Food Future. A Menu of Solutions to Feed nearly 10 Million People by 2050. World Resources Institute. https://www.wri.org/publication/creating-sustainable-food-future.

[2] Nijdam D., Rood T., Westhoek H. (2012). The price of protein: Review of land use and carbon footprints from life cycle assessments of animal food products and their substitutes. Food Policy.

[3] Scarborough P., Appleby P., Mizdrak A., Briggs A.M., Travis R., Bradbury K., Key T. (2014). Dietary greenhouse gas emissions of meat-eaters, fish-eaters, vegetarians and vegans in the UK. Clim. Chang..

[4] Tilman D., Clark M. (2014). Global diets link environmental sustainability and human health. Nature.

[5] Hilborn R., Banobi J., Hall S.J., Pucylowski T., Walsworth T.E. (2018). The environmental cost of animal source foods. Front. Ecol. Environ..

[6] Poore J., Nemecek T. (2018). Reducing food’s environmental impacts through producers and consumers. Science.

[7] ISO (2006). ISO 14040. Environmental Management—Life Cycle Assessment—Principles and Framework.

[8] ISO (2006). ISO 14044. Environmental Management—Life Cycle Assessment—Requirements and Management.

[9] Pelletier N., Audsley E., Brodt S., Garnett T., Henriksson P., Kendall A., Kramer K.J., Murphy D., Nemecek T., Troell M. (2011). Energy intensity of agriculture and food systems. Annu. Rev. Environ. Resour..

[10] Hallström E., Bergman K., Mifflin K., Parker R.W.R., Tyedmers P., Troell M., Ziegler F. (2019). Combined climate and nutritional performance of seafoods. J. Clean. Prod..

[11] FAO (2018). The State of the World Fisheries and Aquaculture (SOFIA).

[12] Robards M.D., Reeves R.R. (2011). The global extent and character of marine mammal consumption by humans: 1970–2009. Biol. Conserv..

[13] Parodi A., Leip A., De Boer I.J.M., Slegers P.M., Ziegler F., Temme E.H.M., Herrero M., Tuomisto H., Valin H., Van Middelaar C.E. (2018). Future foods: Towards a sustainable and healthy diet for a growing population. Nat. Sustain..

[14] Hansen J.C., Deutch B., Odland J.Ø. (2008). Dietary transition and contaminants in the Arctic: Emphasis on Greenland. Int. J. Circumpolar Health.

[15] Government of Greenland (2018). Appendix II in: White Paper on Management and Utilization of Large Whales in Greenland IWC/67/ASW/05.

[16] Hansen J.C. (2000). Grønlandsk kost—En Miljømedicinsk Vurdering (in Danish, Greenlandic Die—An Evaluation from an Environmental Medicine Perspective).

[17] Bjeregaard P., Mulvad G. (2012). The best of two worlds: How the Greenland Board of nutrition has handled conflicting evidence about diet and health. Int. J. Circumpolar Health.

[18] Nutrient Board Greenland (2013). Contamination and Greenlandic Food. In Danish. https://www.google.gl/url?sa=t&rct=j&q=&esrc=s&source=web&cd=1&ved=2ahUKEwjw_82JgeLiAhXrguAKHciCBhAQFjAAegQIAxAC&url=https%3A%2F%2Fwww.peqqik.gl%2F-%2Fmedia%2FFiles%2FMaterialebestilling%2FKontaminant_pjece%2FForurening-og-gr%25C3%25B8nlandsk-mad_DK.pdf%3Fla%3Dda-DK&usg=AOvVaw2g7uPtWzG4AwCqjc-ADYHK.

[19] Nutrient Board Greenland (2019). The 10 Diet Advices. https://www.peqqik.gl/kl-GL/Emner/Livsstil/Kost/GodeRaadOmKost?sc_lang=da-DK.

[20] Freeman M.R.M. (2001). Small-Scale Whaling in North America. http://www.fao.org/docrep/004/Y1290E/y1290e0f.htm#fn1.

[21] Fumagalli M., Moltke I., Grarup N., Racimo F., Bjerregard P., Jørgensen M.E., Korneliussen T.S., Gerbault P., Skotte L., Linneberg A. (2015). Greenlandic Inuit show genetic signatures of diet and climate adaptation. Science.

[22] Government of Greenland (2012). Management and Utilization of Seals in Greenland (“the White Paper”) and Government of Greenland 2015. Data Update to: Management and Utilization of Seals in Greenland (“the white paper”) from 2012.

[23] WWF (2013). Seals in Greenland—An Important Component of Culture and Economy. The Last Ice Area Project..

[24] NAMMCO (2018). Overview of Marine Mammal Hunting Methods including National Regulations, Monitoring/Observation in NAMMCO Member Countries. https://nammco.no/topics/committee-on-hunting-methods/.

[25] EC (2015). EU Regulation 2015/1775. Amending Regulations EC 1007/2009 on Trade in Seal Products, and Repealing Regulation 737/2020.

[26] Baumann H., Tillman A.-M. (2004). The Hitch-hikers guide to LCA.

[27] Hauschild M.Z., Goedkoop M., Guinée J., Heijungs R., Huijbregts M., Jolliet O., Margni M., de Schryver A., Humbert S., Laurent A. (2013). Identifying best existing practice for characterization modeling in life cycle impact assessment. Int. J. Life Cycle Assess..

[28] Finnveden G., Hauschild M.Z., Ekvall T., Guinée J., Heijungs R., Hellweg S., Koehler A., Pennington D., Suh S. (2009). Recent developments in Life Cycle Assessment. J. Environ. Manag..

[29] Leip A., Weiss F., Wassenaar T., Perez-Dominguez I., Fellmann T., Loudjani P., Tubiello F., Grandgirard D., Monni S., Biala K. (2010). Evaluation of the livestock sector’s contribution to the EU greenhouse gas emissions (GGELS) Final Report. Jt. Res. Centre.

[30] Leip A., Billen G., Garner J., Frizzetti B., Lassaletta L., Reis S., Simpson D., Sutton M.A., de Vries W., Weiss F. (2015). Impacts of European livestock production: Nitrogen, sulphur, phosphorous and greenhouse gas emissions, land-use, water eutrophication and biodiversity. Environ. Res. Lett..

[31] IPCC (2014). AR5 Synthesis Report: Climate Change 2014 Fifth Assessment Report. https://www.ipcc.ch/report/ar5/syr/.

[32] Ziegler F., Ritzau Eigaard O., Parker R.W.R., Tyedmers P., Skontorp Hognes E., Jafarzadeh S. (2019). Adding perspectives to: Global trends in carbon dioxide (CO2) emissions from fuel combustion in marine fisheries from 1950–2016. Mar. Policy.

[33] Government of Greenland (2019). Business in Greenland. https://www.businessingreenland.gl/~/media/Fiskeri%20og%20fangst/Hvaler/Havdyrenes%20navne%20oversigt%20DK.pdf?la=da.

[34] Rands M.R., Adams W.M., Bennun L., Butchart S.H., Clements A., Coomes D., Entwistle A., Hodge I., Kapos V., Scharlemann J.P.W. (2010). Biodiversity conservation: Challenges beyond 2010. Science.

[35] Butterworth A., Richardson M. (2014). A review of the animal welfare implications of the Canadian commercial seal hunt. Mar. Policy.

[36] Butterworth A., Richardson M. (2014). A review of the animal welfare implications of the Canadian commercial seal hunt—A response to critique of paper MP13 172. Mar. Policy.

[37] Daoust P.-Y., Hammill M., Stenson G., Caraguel C. (2014). A review of the animal welfare implications of the Canadian commercial seal hunt. Mar. Policy.

[38] NAMMCO Proceedings of the NAMMCO Expert Group Meeting on Hunting Methods for Seals and Walrus.

[39] NAMMCO Proceedings of the NAMMCO Expert Group Meeting on Best Practises in the Hunting and Killing of Seals.

[40] EFSA (2007). 2007. European Food Safety Authority. Scientific Opinion of the Panel on Animal Health and Welfare on a request from the Commission on the Animal Welfare aspects of the killing and skinning of seals. EFSA J..

[41] Hemsworth P.H. (2018). Key determinants of pig welfare: Implications of animal management and housing design on livestock welfare. Anim. Prod. Sci..

[42] Mench J.A. (2018). Advances in Poultry Welfare.

[43] Pedersen L.J., Špinka M. (2017). Overview over commercial pig production systems and their main welfare challenges. Advances in Pig Welfare.

[44] Špinka M. (2017). Advances in Pig Welfare.

[45] ICES (2016). Report of the ICES/NAFO/NAMMCO Working Group on Harp and Hooded Seals (WGHARP). http://www.ices.dk/sites/pub/Publication%20Reports/Expert%20Group%20Report/acom/2016/WGHARP/WGHARP%20Report%20FINAL.pdf.

[46] IUCN (2018). The IUCN Red List of Threatened Species. http://www.iucnredlist.org/.

[47] GRL (2019). Greenlandic Red List 2018. https://natur.gl/raadgivning/roedliste/.

[48] NAMMCO (2019). Website with Information about Atlantic Walrus. https://nammco.no/topics/atlantic-walrus/#1475844586552-bbd974dc-67bc.

[49] Nordborg M., Davis J., Cederberg C., Woodhouse A. (2017). Freshwater ecotoxicity impacts from pesticide use in animal and vegetable foods produced in Sweden. Sci. Total Environ..

[50] Dietz R. (2008). Contaminants in Marine Mammals in Greenland—With Linkage to Trophic Levels, Effects, Diseases and Distribution. Ph.D. Thesis.

[51] UN General Assembly. Document A/69/71 to the Fifteenth Meeting of the United Nations Open-Ended Informal Consultative Process on Oceans and the Law of the Sea, Entitled: “The Role of Seafood in Global Food Security”. https://www.un.org/depts/los/general_assembly/contributions_2015/FAO.pdf.

[52] FAO (2016). Climate Change and Food Security: Risks and Responses.

[53] Vincent A.C.J., Sadovy de Mitcheson Y.J., Fowler S.J., Lieberman S. (2014). The role of CITES in the conservation of marine fishes subject to international trade. Fish Fish..

[54] Bundy A., Heymans J.J., Morissette L., Savenkoff C. (2009). Seals, cod and forage fish: A comparative exploration of variations in the theme of stock collapse and ecosystem change in four Northwest Atlantic ecosystems. Prog. Oceanogr..

[55] Rosing-Asvid A. (2010). Sælerne og Økosystemerne (in Danish, The Seals and the Ecosystems). http://www.natur.gl/fileadmin/user_files/Dokumenter/Raadgivning/2010_Notat_om_saelerne_og_oekosystemerne.pdf.

[56] NAMMCO Proceedings of the 25th Meeting of the Council.

[57] NAMMCO (2019). Overview of Marine Mammal Hunting Methods Inc. National Regulations, Monitoring/Observation in Nammco Member Countries (update 08/2019). https://nammco.no/topics/other_documents/.

[58] Government of Greenland (2017). Seal Event in Brussels on QR-Code.

[59] Rasmussen M.Y. (2018). Subsistence? A Critical Analysis of the EU Protection of Indigenous Peoples Rights through the Case of the EU Seal Regime. Master’s Thesis.

[60] COM (2012). Final 494 Communication on Blue Growth—Opportunities for Marine and Maritime Sustainable Growth 13.9.2012 Brussels.

[61] High North Alliance (2008). On the Carbon Footprint of Whale Meat.

[62] Behaderovic D., Berglund M. (2019). Climate Footprint of Game. Orienting Study about Venison and Klimatavtryck av viltkött. Orienterande Studie om Dovhjort och Vildsvin.

[63] Saxe H. (2015). Is Danish Venison Production Environmentallly Sustainable?.

